# ReLU, Sparseness, and the Encoding of Optic Flow in Neural Networks

**DOI:** 10.3390/s24237453

**Published:** 2024-11-22

**Authors:** Oliver W. Layton, Siyuan Peng, Scott T. Steinmetz

**Affiliations:** 1 Department of Computer Science, Colby College, Waterville, ME 04901, USA; siyuanpeng@microsoft.com; 2 Microsoft Corporation, Redmond, WA 98052, USA; 3 Center for Computing Research, Sandia National Labs, Albuquerque, NM 87123, USA; ststein@sandia.gov

**Keywords:** optic flow, self-motion, heading, sparseness, sparsity, activation function, neural network, CNN, deep learning

## Abstract

Accurate self-motion estimation is critical for various navigational tasks in mobile robotics. Optic flow provides a means to estimate self-motion using a camera sensor and is particularly valuable in GPS- and radio-denied environments. The present study investigates the influence of different activation functions—ReLU, leaky ReLU, GELU, and Mish—on the accuracy, robustness, and encoding properties of convolutional neural networks (CNNs) and multi-layer perceptrons (MLPs) trained to estimate self-motion from optic flow. Our results demonstrate that networks with ReLU and leaky ReLU activation functions not only achieved superior accuracy in self-motion estimation from novel optic flow patterns but also exhibited greater robustness under challenging conditions. The advantages offered by ReLU and leaky ReLU may stem from their ability to induce sparser representations than GELU and Mish do. Our work characterizes the encoding of optic flow in neural networks and highlights how the sparseness induced by ReLU may enhance robust and accurate self-motion estimation from optic flow.

## 1. Introduction

Optic flow is the pattern of motion that arises on the camera sensor as the camera moves with respect to its surrounding environment (self-motion). It contains rich visual information about the speed, direction, and distance of the camera relative to objects, surfaces, and edges in the visual scene [1]. For this reason, optic flow is used to support autonomous navigation in mobile robotic systems, such as micro unmanned aerial vehicles (MAVs) [2,3,4,5]. By virtue of their small size, MAVs operate with extremely limited payloads and power budgets. Despite these constraints, on-board hardware is capable of extracting and processing optic flow to support navigation, even at high speeds. For example, Escobar-Alvarez and colleagues developed a MAV that relies on optic flow to fly through cluttered indoor and outdoor environments while avoiding obstacles and reaching speeds of 19 m/s [6]. While optic flow extraction requires only a single camera, the inclusion of LiDAR sensors or a second camera (stereo cameras) provides additional information about the depth of objects in the visual scene. This has been shown to improve estimates of self-motion and the position of the mobile system in its environment [7]. However, these extra sensors add substantial weight and bulk, which hinders their suitability in MAV applications. Advances in optic flow-based navigation could improve the capabilities of MAV systems without adding to their physical size or decreasing their endurance.

**Figure 1:**
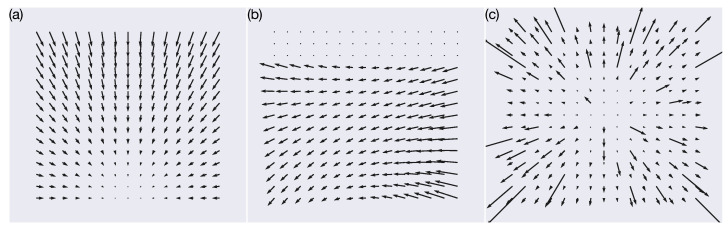
Sample optic flow fields generated with different simulated camera self-motion through different visual environments. (**a**) Backward translation at 1 m/s at −180° azimuth and 45° elevation away from a frontoparallel wall positioned 4 m in front of the camera. (**b**) Combination of backward translation at 1 m/s (−180° azimuth, 45° elevation) with respect to a ground plane and 5°/s yaw rotation. The camera is 10 m above the ground plane and is oriented 30° downward. (**c**) Forward translation at 1 m/s at 0° azimuth and 0° elevation through a 3D dot cloud.

To that end, a primary goal of the present article is to characterize the accuracy and robustness of self-motion estimates from optic flow collected under controlled conditions in a number of important navigation scenarios. Mathematically, self-motion is defined as two vectors: the 3D translation ($\vec{T}=\left(T_{x},T_{y},T_{z}\right)$) and rotation ($\vec{R}=\left(R_{x},R_{y},R_{z}\right)$) vectors that specify the instantaneous displacement and rotation of the camera, respectively. The present paper focuses on the direction of translation, so we parameterize translation as a pair of azimuth and elevation angles. Figure 1a shows the optic flow generated by a camera translating away from a frontoparallel wall at 1 m/s straight-backward (−180° azimuth) and upward (45° elevation). Figure 1b depicts a combination of translation and rotation in the case of straight-backward translation combined with 5°/s rotation about the vertical y-axis (i.e., $R_{x}=R_{z}=0$).

**Figure 2:**
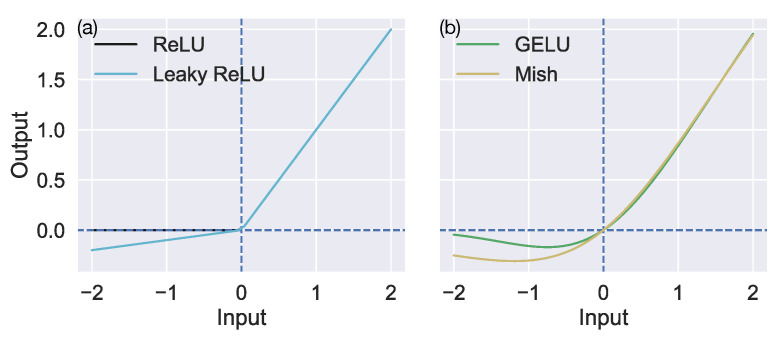
Neural network activation functions examined in the present article. (**a**) The rectified linear unit (ReLU) and leaky ReLU activation functions. (**b**) The Gaussian error linear unit (GELU) and Mish activation functions. The y-axis shows the output of the neuron after applying an activation function to the net input indicated on the x-axis.

Over the past decade, deep convolutional neural networks (CNNs) have made major strides toward giving rise to accurate and robust self-motion estimation from optic flow [8,9,10,11,12,13,14,15,16,17]. These algorithms typically estimate the translation ($\vec{T}$) and rotation ($\vec{R}$) of the camera based on signals derived from optic flow input (Figure 1) or sequences of images captured by the camera, processed through a cascade of neural layers. Virtually all these CNNs use the rectified linear unit (ReLU) activation function to transform the neural signal as it passes through the hidden layers of the network. ReLU applies a nonlinear gating operation to input signals, allowing nonnegative inputs to pass through the layer as-is while zeroing out negative inputs (Figure 2a). The positive portion of ReLU provides a simple, computationally efficient gradient of 1, which can be used during training to update a neuron’s weights and propagate gradients backward to previous layers of the network via the backpropagation algorithm. However, gradients cannot flow backward through a ReLU neuron when it receives negative input since the gradient is 0. This may give rise to a phenomenon known as the Dying ReLU where the weights of a neuron do not allow it to activate for any input, and, because the gradient is 0, the weights cannot be updated to potentially change this behavior [18]. Maas and colleagues introduced the piecewise linear leaky ReLU activation function [19], which is identical to ReLU except that it backpropagates a small constant gradient for negative net inputs to prevent a neuron from becoming a dead neuron (Figure 2a).

Despite the prevalence of dead neurons, Glorot and colleagues showed that multi-layer perceptron (MLP) neural networks configured with ReLU garner better accuracy on image datasets than MLPs configured with the other activation functions while generating incredibly sparse patterns of activations across the network [20]. Indeed, only ≈15–30% of neurons in the network activated to inputs. A sparse representation has the potential advantage of being invariant to noise and other small changes in the input [11,20,21]. By contrast, unimportant small fluctuations in an input may alter most components of a dense code that involves every neuron, making decoding from the network more challenging. Sparse codes may also increase the separability between dissimilar inputs because different features could recruit mostly non-overlapping neural subpopulations [22,23,24]. This could potentially enhance the speed and accuracy of inference [25,26].

The aim of the present study is to investigate the extent to which ReLU induces a sparse encoding in MLPs and CNNs that are optimized to estimate self-motion from optic flow and examine whether this representation offers superior accuracy, robustness, and generalization in important self-motion scenarios. We focused on MLPs because they induce sparse encodings of image and text data [20] and on CNNs because of their prevalence and success at estimating self-motion from optic flow [8,9,10,11,12,13,14,15,16,17]. To evaluate ReLU’s contributions to the neural representation of optic flow, we trained MLPs and CNNs with one of three alternative activation functions that do not suffer from the dying ReLU problem and may yield less sparse representations: leaky ReLU, Gaussian error linear unit (GELU) [27], and Mish [28]. Leaky ReLU provides insight into how small negative activations for negative net inputs, its primary distinction from ReLU (Figure 2a), influence the encoding. We included GELU and Mish because they are associated with high-performing neural networks. GELU is widely used in transformers and recent state-of-the-art CNNs [29], while Mish has demonstrated higher accuracy than leaky ReLU on large image datasets. Like leaky ReLU, both GELU and Mish allow small negative activations, but they differ in being smooth mathematical functions (Figure 2b). The present study builds upon our existing work [17,30] that explores the impact of nonnegativity and L1 sparseness constraints on the encoding of optic flow in CNNs.

## 2. Materials and Methods

We utilize optic flow datasets that we developed for our prior study [17,30] to train and evaluate the neural networks, which we describe below for completeness (see specifications in Table 1). After describing these datasets, we present our model training protocol (Table 2) and hyperparameters (Table 3 and Table 4). We end the section with a description of our analyses, which focus on the robustness of model predictions in novel self-motion scenarios, the sparseness of the optic flow encoding, and neural tuning to translational and rotational optic flow.

### 2.1. Optic Flow Datasets

Table 1 summarizes the datasets generated in [17,30] and used in the present study. We trained the neural networks on a dataset (TR360) that consists of optic flow stimuli generated from randomly sampled translation and rotation directions along the 3D unit sphere (i.e., all possible 3D directions are sampled uniformly). We scaled each direction into the range of typical human walking speeds (T speed in Table 1). The visual scene consists of either a frontoparallel plane (wall; Figure 1a) or ground plane (Figure 1b). Each sample in the datasets corresponds to a vector field, capturing the optic flow at a specific time instant, as the observer moves with a 3D translational velocity $\vec{T}=\left(T_{x},T_{y},T_{z}\right)$ and rotational velocity $\vec{R}=\left(R_{x},R_{y},R_{z}\right)$. We simulated an observer with a 90° field of view and assume that the projection of points from the visual scene onto the camera occurs using a pinhole camera model with focal length f=1 cm [31]. Following [17,32], we compute the optic flow on an evenly spaced 15 × 15 grid of points within the image plane of the camera. The *x* and *y* coordinates of these points both span [−f,f], the central portion of the image plane that falls within the simulated 90° field of view [31,32]. Given a translation $\left(\vec{T}\right)$ vector, a rotation vector $\left(\vec{R}\right)$, and the 2D grid of sample points on the image plane (x,y), we evaluated the instantaneous optic flow $\left(\dot{x},\dot{y}\right)$ [33]:

(1) $$\left(\begin{array}{c} \dot{x}\\ \dot{y} \end{array}\right)=\frac{1}{Z(x,y)}\left(\begin{array}{ccc} -f & 0 & x\\ 0 & -f & y \end{array}\right)\left(\begin{array}{c} T_{x}\\ T_{y}\\ T_{z} \end{array}\right)+\frac{1}{f}\left(\begin{array}{ccc} xy & -\left(f^{2}+x^{2}\right) & fy\\ \left(f^{2}+y^{2}\right) & -xy & -fx \end{array}\right)\left(\begin{array}{c} R_{x}\\ R_{y}\\ R_{z} \end{array}\right)$$

In Equation (1), Z(x,y) refers to the depth of the point in the world that projects to point (x,y) on the image plane. For the frontoparallel plane scene, Z(x,y) is set to the relative depth of the plane (see Table 1). For the ground plane scene, $Z\left(x,y\right)=Z\left(y\right)=hf/\left(ycos\left(\alpha \right)+fsin\left(\alpha \right)\right)$, where *h* is the height of the ground plane relative to the camera sensor, *f* is the camera focal length, and α is the vertical offset angle of the gaze relative to the horizontal axis. We fixed the *h* and α parameters to the values set by existing studies that use the TR360 dataset [17,30,32] to facilitate comparison. We set h=−10 m (i.e., the ground plane is 10 m below the camera) and α=−30 (i.e., the camera orientation is directed 30° below the horizon). The dimensions of each optic flow dataset are (N,15,15,2), where *N* represents the number of data samples, and 2 refers to the horizontal and vertical portion of each optic flow vector $\left(\dot{x},\dot{y}\right)$.

Following [17,30], we trained each model using the TR360 dataset and reserved the other datasets to test generalization on novel optic flow patterns. To generate the sample sizes indicated in Table 1, unless otherwise specified, we systematically crossed the independent variables and replicated these conditions until we reached the total number of samples. For instance, we produced the 6030 TR360 samples for the frontoparallel environment by combining T speed (3 levels), R speed (3 levels), and depth (5 levels), resulting in 45 unique combinations. We replicated this process 134 times to achieve the 6030 samples. Each time we generated a sample, we drew random values for the other variables (in this case, T and R directions) to ensure that no duplicate samples appeared in the dataset. The TR360 dataset is evenly split with respect to visual environment: half of the samples simulate self-motion toward the frontoparallel plane, while the other half simulate motion over the ground plane. Before model training and subdividing the datasets into train, validation, and test splits, we randomly shuffled the order of the data samples.

We test the generalization of the fitted models by estimating self-motion during simulated self-motion through a 3D dot cloud (TR360Cloud), a novel environment not experienced during training (see Table 1). Figure 1c shows an example of an optic flow field in the TR360Cloud dataset. The remaining datasets listed in Table 1, TestProtocolT and TestProtocolR, represent collections of optic flow diagnostic patterns that we use to identify the self-motion direction that optimally activates every model neuron. In these datasets the azimuth and elevation angles systematically vary on an evenly spaced grid (11.25° steps). TestProtocolT only contains translational self-motion, and TestProtocolR only contains rotational self-motion.

#### Robustness Tests

We test the robustness of the fitted neural network models in three novel self-motion scenarios: optic flow corrupted with noise, sparse optic flow, and self-motion in the presence of an independently moving object. All three scenarios involve perturbations that we introduced to the TR360 test set.

For the noise scenario, we shuffled the position of a proportion of optic flow vectors within the 15 × 15 grid. We maintained a consistent noise proportion by shuffling the same fraction of vectors in each test sample, with different vectors being randomly selected for repositioning each time. We created 11 variants of the TR360 test set that use the following noise proportions: [0.0, 0.1, …, 1.0].

We implemented the sparse optic flow scenario similarly by randomly setting the magnitude of a proportion of optic flow vectors in each test sample to 0. We used the following sparseness values: [0.0, 0.1, 0.2, 0.3, 0.4, 0.5, 0.6, 0.7, 0.8, 0.9, 0.95, 0.99].

For the independently moving object scenario, we introduced a pattern of motion to the optic flow field that corresponds to the moving object. This pattern replaces a square portion of the optic flow field with an array of uniform motion vectors. We assigned the direction of every vector belonging to the moving object to a single random angle (0–360°), which we generated anew for each test sample. We set the speed of each object vector to the mean speed of the vectors in the optic flow field before the introduction of the moving object. The resulting optic flow pattern arises during self-motion in the presence of an independently moving object that maintains a constant depth with respect to the observer [31,34,35]. We created 5 variants of the TR360 test set, each with a constant object size (length and width): either 1, 2, 3, 6, or 12 pixels. We positioned the object randomly within each test sample.

### 2.2. Neural Networks

#### 2.2.1. Architecture

**Figure 3:**
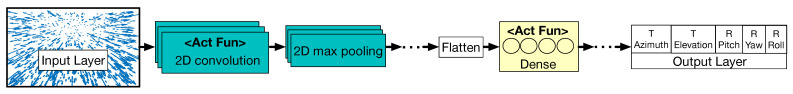
Overview of the CNN and MLP network architecture. The CNN architecture begins with one or more convolutional and max pooling layer stacks. The max pooling layers that reduce the spatial resolution of the optic flow signal. The representation in the final max pooling layer is flattened into a 1D vector, which is passed through one or more densely connected layers. As described in the main text, we created CNN and MLP variants that apply one of the following activation functions in both the convolutional and dense layers: ReLU, leaky ReLU, GELU, or Mish. We schematize where in the network the choice of one of these activation functions is applied with <Act fun>. The output layer contains five neurons, corresponding to the parameters that describe the camera’s self-motion: the azimuth and elevation of observer translation, along with the pitch, yaw, and roll components of observer rotation. The network is trained to minimize a cosine loss function of the translation azimuth angle due to its circularity. Mean squared error (MSE) is used for the other variables. The MLP differs from the CNN in the lack of convolutional and max pooling stages (shown in teal).

We configured CNNs using the same architectural building blocks as [17,30]. Figure 3 schematizes the overarching structure of the network. The first convolutional layer applies 2D spatial convolution (stride: 1, padding: ‘same’) to the optic flow input (shape: (*N*, 15, 15, 2)). We then apply a selected activation function (<Act fun>, detailed below) and process the resulting activations with 2D max pooling. The network may contain additional interleaved combinations of such 2D convolutional and max pooling layers. After the last 2D max pooling layer, the activations are flattened and passed to one or more densely connected hidden layers configured with the same activation function used in the earlier convolutional layer(s). The output layer consists of five neurons, each corresponding to the self-motion parameters: translational azimuth, translational elevation, rotational pitch ($R_{x}$), rotational yaw ($R_{y}$), and rotational roll ($R_{z}$). We normalized each label to the range [−0.5, 0.5] before training. We trained the CNN to minimize the total loss from estimating these five self-motion variables. Mean squared error (MSE) loss is used for translational elevation and the three rotational self-motion parameters. We used a cosine loss function for translational azimuth due to its circular range of [−180, 180]°:

(2) $$L_{circ}=\frac{1}{B}\sum_{i=1}^{B}\frac{1}{2}\left[1-\cos\left(\pi \left(y_{i}-{\hat{y}}_{i}\right)\right)\right]$$

In the above equation, *B* refers to the mini-batch size, $y_{i}$ refers to the T azimuth label for sample *i* on the normalized scale, and ${\hat{y}}_{i}$ is the normalized predicted T azimuth value. A key characteristic of the cosine loss function (Equation (2)) is that it assigns the same cost to predictions that differ by 360°, which are equally valid. The loss is 0 when the network prediction matches the label. The cost reaches 1 when the true and predicted values differ by 180° (e.g., predicting straight-ahead when the actual movement is straight-back). We used the Adam optimizer with default settings, except as noted below.

We initialized the weights in each network using the Glorot uniform method, the TensorFlow default [36,37]. This means that the initial value of each weight is drawn from the following uniform distribution:

(3) $$U\left[-\sqrt{\frac{6}{F_{in}+F_{out}}},\sqrt{\frac{6}{F_{in}+F_{out}}}\right]$$

In Equation (3), $F_{in}$ and $F_{out}$ refer to the number of units in the previous and current layer, respectively.

#### 2.2.2. Model Variants

We created several variants of CNNs and MLPs to investigate the influence of the activation function on the accuracy of self-motion estimates and the encoding of optic flow. The MLPs possess the same architecture as the CNN shown in Figure 3, except for a lack of the convolution and max pooling layers (no teal boxes). We configured each model variant to use a distinct activation function in the convolutional and dense layers (<Act fun> in Figure 3): ReLU, leaky ReLU, GELU, or Mish. We created 8 networks in total: 4 CNNs (CNN_RELU, CNN_LEAKY_RELU, CNN_GELU, CNN_MISH) and 4 MLPs (MLP_RELU, MLP_LEAKY_RELU, MLP_GELU, MLP_MISH).

The ReLU activation function is defined as

(4) f(x)=max(x,0)

where *x* in Equation (4) refers to the net input to a model neuron.

The leaky ReLU activation function [19] is defined as

(5) $$f\left(x\right)=\left\{\begin{array}{cc} x & \mathrm{if}\;x>0,\\ \alpha x & \mathrm{if}\;x\leq 0 \end{array}\right.$$

where the α hyperparameter controls the slope of the activation function for negative inputs (Figure 2a). We included α in our hyperparameter search (Table 2), and α=0.1 was selected as the optimal value for both models that use the leaky ReLU (CNN_LEAKY_RELU, MLP_LEAKY_RELU).

The GELU activation function [27] is defined as

(6) $$f\left(x\right)=x\cdot \frac{1}{2}\left(1+\mathrm{erf}\left(\frac{x}{\sqrt{2}}\right)\right)$$

where erf(*x*) is the cumulative distribution function of the standard normal distribution.

The Mish activation function [28] is defined as

(7) $$f\left(x\right)=x\cdot \tanh\left(Log\left(1+e^{x}\right)\right)$$

We optimized the architecture and hyperparameters of each neural network variant independently to find the configuration that yields the lowest validation loss (i.e., the most accurate self-motion estimates). We searched for the hyperparameters listed in Table 2 over several thousand iterations. Each search involved fitting each model to the TR360 training set and recording the hyperparameters that yield the smallest validation loss summed across the 5 output neurons. On each search iteration, we randomly selected values for each hyperparameter from the ranges enumerated in Table 2. These ranges are informed by previously performed hyperparameter searches involving CNNs with the ReLU activation functions on the TR360 dataset [17,30], which showed no improvement in the validation loss with additional units and layers.

#### 2.2.3. Training Protocol

We developed the neural networks using TensorFlow 2.11 and Python 3.10 [37]. We trained the models on an NVIDIA GeForce RTX 4090 GPU and a Microsoft Windows 11 machine. When training the models, we fixed the mini-batch size to 64 and utilized early stopping (patience: 60 epochs). Table 3 shows the optimized hyperparameter values for the CNNs, and Table 4 shows those for the MLPs. Table 5 shows a comparison of the number of parameters in each network.

### 2.3. Analyses

Here we describe analyses that we performed on the trained neural network models to investigate how they encode optic flow.

#### 2.3.1. Population and Lifetime Sparseness Metrics

We used the population and lifetime sparseness metrics to quantify the sparseness of the activations within each network layer of the models [38]. Both metrics range between 0 and 1 and are computed with the following equation:

(8) $$s=\left(1-\frac{1}{N}\frac{{\left(\sum_{i=1}^{N}r_{i}\right)}^{2}}{\sum_{i=1}^{N}r_{i}^{2}}\right)/\left(1-\frac{1}{N}\right)$$

For population sparseness, $r_{i}$ in Equation (8) denotes the activation of neuron *i* to a specific optic flow pattern, and *N* denotes the number of neurons in the layer. This computation yields a scalar value for each layer of the models. Values close to 1 indicate that few neurons activate to the pattern, indicating a sparse code. For lifetime sparseness, $r_{i}$ in Equation (8) denotes the activation of a neuron to optic flow pattern *i*, and *N* denotes the number of optic flow patterns in the dataset. Values close to 1 indicate that the neuron rarely activates to samples in the dataset, also indicating a sparse code. We averaged the values among neurons belonging to a single network layer.

#### 2.3.2. Weight Sparseness Index

We use the Sparseness Index (*S*) of [39] to quantify the sparseness of the weights of a particular model layer. To compute *S*, we first take the absolute value of the weights of a layer and flatten them into a 1D array. Next, we compare values in this weight array to 200 thresholds, equally spaced in between the minimum and maximum absolute weight values. We compute the proportion of weights that are larger than each threshold value. From the resulting proportions, we compute the area under the curve (AUC) using the trapezoid rule. The Sparseness Index (*S*) is defined as *S*

(9) S=1−2AUC.

*S* values close to 0 indicate that the weights follow a uniform distribution and densely span the minimum and maximum values. *S* values close to 1 correspond to a sparse distribution where few weights are nonzero.

#### 2.3.3. Translation and Rotation Tuning Preferences

To gain more insight about the encoding of optic flow in the neural networks, we characterized the translational and rotational optic flow preferences of individual model neurons. The translational (rotational) preference of a neuron defines the self-motion direction (azimuth and elevation angle) that yields the maximal activation when assessed using purely translational (rotational) optic flow. We determined each neuron’s preference using a pair of diagnostic translational (TestProtocolT) and rotational (TestProtocolR) optic flow datasets within which the direction of translation and rotation, respectively, of the observer systematically varies (see Table 1). We computed the activation of single neurons to all the optic flow samples in either the TestProtocolT or TestProtocolR datasets and then applied the population vector decoding method [32,40] to determine the translational and rotational preferences. The preference of the neuron is the direction that corresponds to the weighted sum between the known translational or rotational labels and the activation to each corresponding optic flow pattern.

### 2.4. Software Accessibility

We implemented and simulated the neural networks in Python using the NumPy [41], SciPy [42], Pandas [43], Seaborn [44], and TensorFlow [37] libraries. The trained models and datasets are available on Hugging Face: https://huggingface.co/collections/OWLab/optic-flow-cnns-mlps-15x15-673f74ad3d22cacbcc19c39c (accessed on 21 November 2024). Code is available on GitHub: https://github.com/owlayton/DL-ActFuns-Acc-SelfMotion-Release (accessed on 21 November 2024).

## 3. Results

Our goal was to investigate how the ReLU activation function influences the accuracy with which CNNs estimate self-motion from optic flow, affects their ability to generalize to novel conditions, and shapes the neural encoding. To that end, we optimized CNNs to estimate the observer’s visual translation and rotation on a 6030-sample optic flow dataset (TR360) composed of combinations of 3D linear translations and rotations (see Table 1). We created 4 CNN variants, each of which implements a different activation function: ReLU, Leaky ReLU, GELU, and Mish (CNN_RELU, CNN_LEAKY_RELU, CNN_GELU, CNN_MISH). We optimized each variant independently of the others in order to determine the optimal hyperparameters and network structure that are tailored to each activation function. To better understand how the encoding of optic flow depends on the convolution operation in the early layers and may interact with the selected activation function, we compared the CNNs to MLPs, which possess the same architecture except that they lack the convolution and max pooling layers (Figure 3). As with the CNNs, we created 4 MLP variants that possess different activation functions (MLP_RELU, MLP_LEAKY_RELU, MLP_GELU, MLP_MISH).

We begin the section with a comparison of the accuracy with which the neural networks estimate self-motion from optic flow. We subsequently analyze how well the models estimate self-motion in novel scenarios not encountered during training (i.e., generalization and robustness). Next, we examine the relationship between sparseness in the optic flow encoding and generalization accuracy. We end the section with a characterization of how model neurons encode optic flow with respect to the directions of translation and rotation that yield the greatest activation.

### 3.1. Accuracy of Self-Motion Estimation

**Figure 4:**
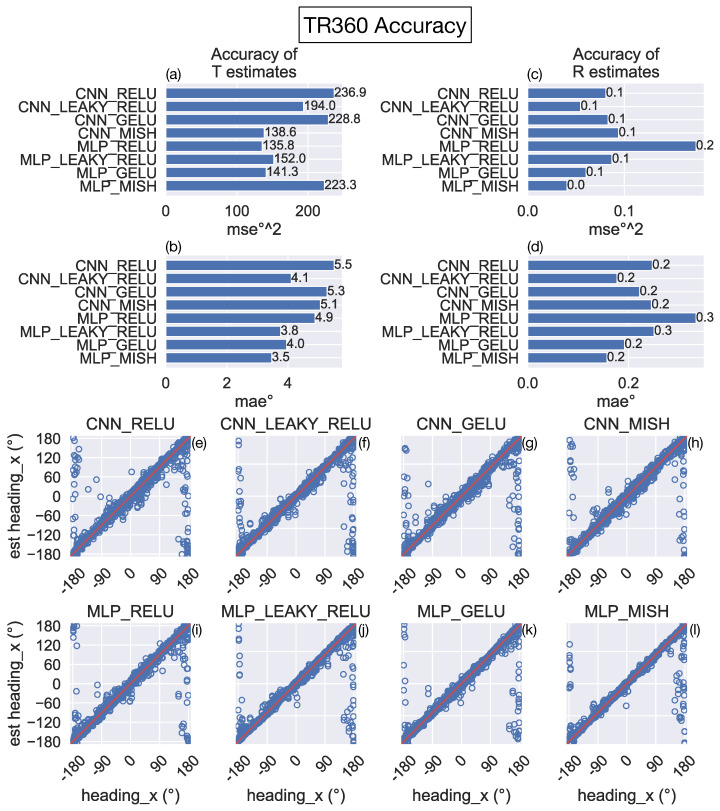
Test accuracy of the neural networks on the TR360 dataset. (**a**,**c**) MSE of network estimates of translational (T) and rotational (R) self-motion from optic flow (**b**,**d**) mean absolute error (MAE) of networks estimates of the T and R self-motion from optic flow. (**e**–**h**) Scatter plots depict the estimate (y-axis) corresponding to each true translational azimuth label (x-axis; “heading_x”) produced by each CNN variant. Each red diagonal line coincides with estimates that match the true label (no error). (**i**–**l**) Same format as the row above, but for the MLPs. In the depicted coordinate system, ±180° both refer to straight-backward self-motion.

We determined the accuracy with which the neural networks estimate the 3D translation and rotation of the camera on the TR360 test set, which contains 3015 novel optic flow samples that the models did not encounter during training. Figure 4a–d shows that the CNNs and MLPs estimate translational and rotational self-motion accurately, regardless of the network architecture and activation function. All the networks estimated camera translation within 5.5° MAE (236.9°^2^ MSE) and rotation within 0.3°/s MAE (0.2°^2^/s^2^ MSE). Figure 4e–l show the individual predictions of the translation azimuth angle from each network. Predictions generally fall close to their true values (unity line), except there is more variability for ±180 azimuths (i.e., backward translation).

#### Generalization: TR360Cloud

We evaluated how well the 8 models trained on the TR360 dataset generalize to optic flow generated from simulated self-motion through a 3D dot cloud (TR360Cloud; Figure 1c), an environment not used in training. Apart from the visual scene, the self-motion characteristics were identical to those in the TR360 dataset (Table 1).

**Figure 5:**
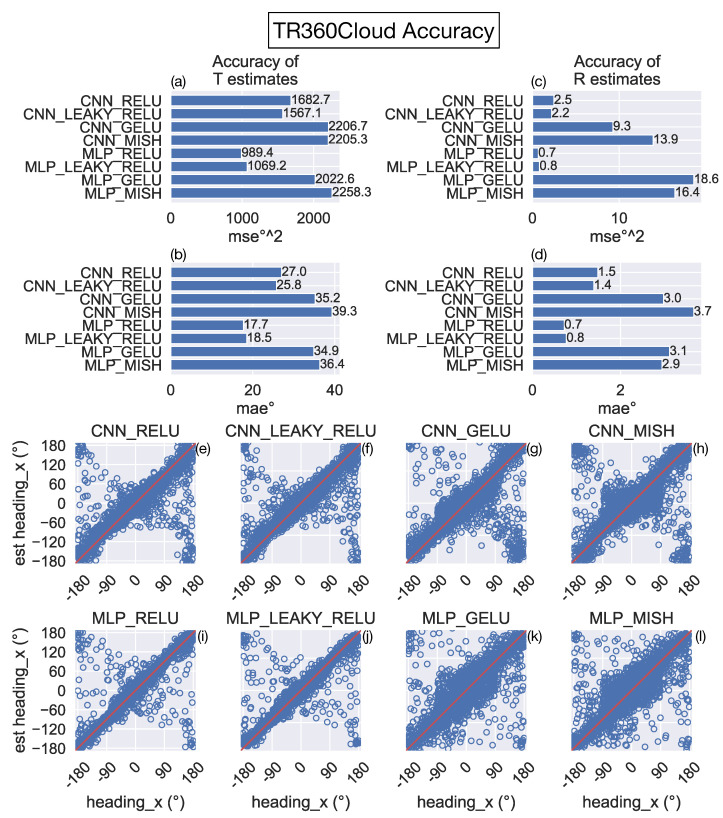
Test accuracy on the TR360Cloud optic flow dataset achieved by the CNN and MLP models trained on a different dataset (TR360). Same format and conventions as Figure 4.

Figure 5a–d show that the mean error induced when estimating the translational and rotational self-motion of the camera is considerably larger overall than that obtained on the TR360 test set across all models (Figure 4). Interestingly, models with the ReLU and leaky ReLU activation functions garner substantially lower error in their estimates. For example, the MLP_RELU and MLP_LEAKY_RELU models yield approximately 50% of the MAE on translation estimates and 25% of the MAE on rotation estimates compared to the MLP_GELU and MLP_MISH models. The improvement with the ReLU and leaky ReLU activation functions occurs for both CNN and MLP architectures. The increase in accuracy is visually apparent from scatter plots showing the true and predicted translational azimuth angle for individual samples (compare Figure 5e,f with Figure 5g,h, Figure 5i,j and Figure 5k,l). In sum, these simulations show that networks that use the ReLU and leaky ReLU activation functions generalize better to the novel 3D dot cloud environment than the networks that use GELU and Mish.

### 3.2. Robustness

Next, we investigated the robustness of model estimates in challenging scenarios in which we introduced perturbations to TR360 optic flow test samples. Our goal was to determine whether certain neural network architectures and activation functions provide greater resilience to disruptions in the optic flow field. We focus on three tests of robustness: the introduction of noise to the optic flow field, sparse optic flow, and the presence of an independently moving object.

#### 3.2.1. Noise

**Figure 6:**
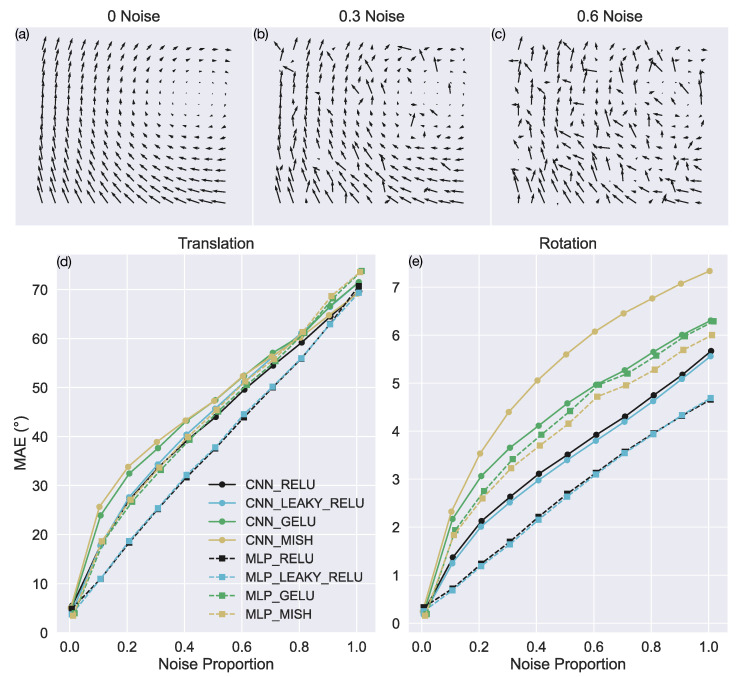
Accuracy of self-motion estimates when noise is added to the TR360 test optic flow samples. (**a**–**c**) Example optic flow fields with 0%, 30%, and 60% noise, respectively. (**d**,**e**) MAE in estimating translational and rotational self-motion parameters, respectively, when the optic flow contains different proportions of noise (x-axis).

In this test, we generated versions of the TR360 test set wherein a certain proportion of optic flow vectors in each optic flow sample are randomly repositioned (“noise vectors”). Figure 6a–c show the same optic flow sample with 0%, 30%, and 60% noise levels, respectively. When an optic flow sample has 100% noise, all the optic flow vectors are spatially shuffled. Each version of the TR360 test set contains a constant proportion of noise in each sample (see Section 2).

Figure 6d,e show the average error in model translational and rotational self-motion estimates, respectively, for various amounts of noise. For translational self-motion estimates, MLP_RELU and MLP_LEAKY_RELU yield the lowest error across most noise levels (Figure 6d). In the case of intermediate levels of noise, the MAE in these networks is ≈7.5° lower than the CNNs with the same activation functions (CNN_RELU and CNN_LEAKY_RELU) as well as the MLP_GELU and MLP_MISH models. The MLP_RELU and MLP_LEAKY_RELU models also garner ≈10° lower error than the CNNs with GELU and Mish. There are less pronounced differences among the models for very low and high levels of noise.

Consistent with their mean accuracy when estimating translational self-motion, the MLP_RELU and MLP_LEAKY_RELU models yield the lowest MAE across all nonzero noise levels when estimating rotational self-motion. The CNN_RELU and CNN_LEAKY_RELU models yield the next lowest MAE, with ≈1° greater MAE than MLP_RELU and MLP_LEAKY_RELU. The networks with GELU and Mish garner the greatest error.

#### 3.2.2. Sparse Optic Flow

**Figure 7:**
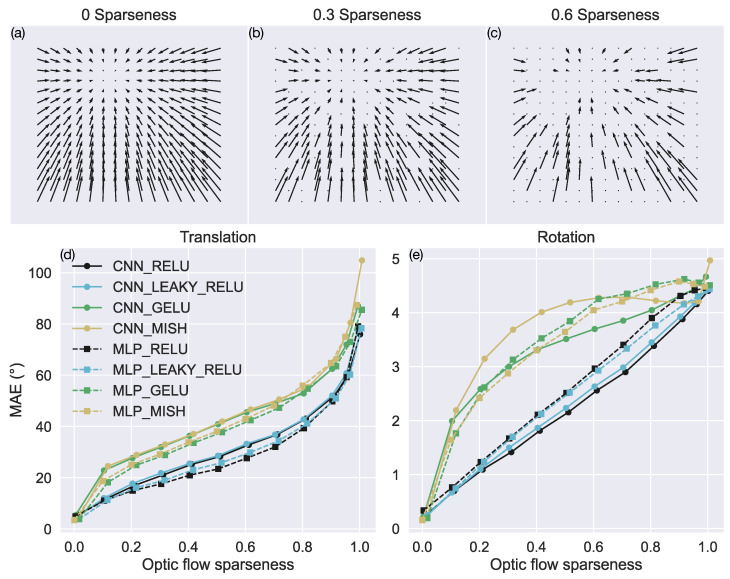
Accuracy of self-motion estimates when motion vectors are removed from the TR360 test optic flow samples. (**a**–**c**) Example optic flow fields with 0%, 30%, and 60% sparseness, respectively. (**d**,**e**) MAE in estimating translational and rotational self-motion parameters, respectively. The x-axis indicates the degree of sparseness within each optic flow sample.

We assessed the impact of sparse optic flow, which refers to the presence of only a fraction of the motion vectors from the original TR360 test set, on self-motion estimates. We followed a similar paradigm to the one used in the noise test and created multiple versions of the TR360 test set, each of which removes a fixed proportion (0–99%) of motion vectors from optic flow samples. Figure 7a–c depict optic flow samples with 0%, 30%, and 60% sparseness.

Figure 7d,e show that the models that employ the ReLU and leaky ReLU activation functions (CNN_RELU, CNN_LEAKY_RELU, MLP_RELU, MLP_LEAKY_RELU) produce substantially lower mean error for both translation and rotation than the models that employ GELU and Mish. For intermediate levels of optic flow sparseness, the ReLU and leaky ReLU networks produce ≈40% less MAE in their translational and rotational self-motion estimates. All the models perform poorly when the optic flow is extremely sparse (≥90%).

#### 3.2.3. Independently Moving Objects

**Figure 8:**
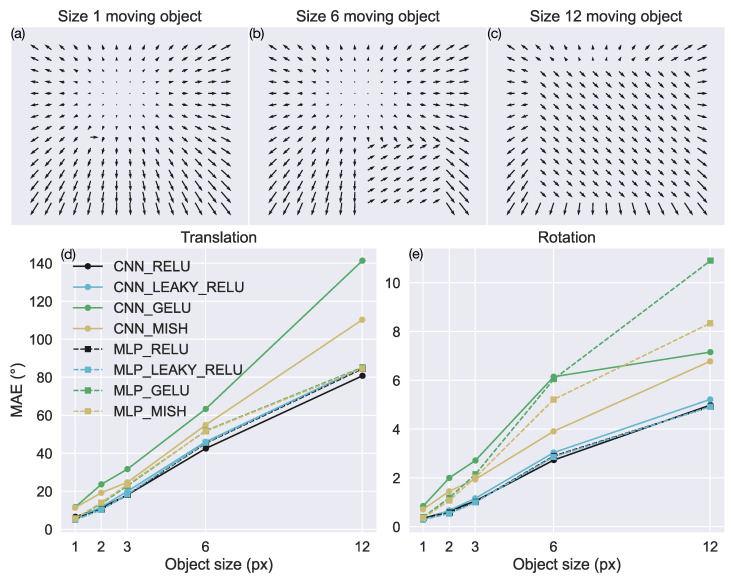
Accuracy of self-motion estimates when the optic flow contains the motion due to an independently moving object. (**a**–**c**) Example optic flow fields with a Size 1 (1 × 1 pixels), Size 6 (6 × 6 pixels), and Size 12 (12 × 12 pixels) region of motion induced by the moving object, respectively. (**d**,**e**) MAE in estimating translational and rotational self-motion parameters, respectively. The x-axis indicates the size of the moving object in the optic flow field.

Under naturalistic conditions, the presence of an object that moves independently of the camera complicates the task of self-motion estimation since the object creates a localized pattern of motion in the optic flow field that is not only determined by the scene-relative movement of the camera. In this test, we introduced motion caused by an independently moving object to the test TR360 optic flow fields to assess the robustness of the neural network models. For simplicity, we implement the moving object by replacing the scene-relative motion where the object appears in the optic flow field. This scenario corresponds to a moving object that maintains a constant depth with respect to the camera [16,31,34,45,46]. Figure 8a shows a case where a small moving object creates a single (1 × 1) rightward motion vector in the optic flow field. Notice how the direction and speed of the object’s motion conflict with the surrounding slow-motion vectors directed down-and-leftward. Figure 8b shows an example with a moderately sized object that replaces a 6 × 6 portion of the optic flow with discrepant motion. The large moving object in Figure 8c replaces a 12 × 12 region of optic flow and thereby removes most of the motion that is consistent with the scene-relative self-motion of the camera.

Figure 8d,e show the accuracy achieved by each model when samples in the TR360 test set contain square moving objects of different sizes. As the size of the moving object increases, the accuracy garnered by the models decreases. Consistent with the sparse optic flow test, CNNs and MLPs with the ReLU and leaky ReLU yield the lowest error in virtually every condition. The neural network architecture of these models exerts little impact on the accuracy.

Taken together, our simulations reveal that neural networks configured with the ReLU and leaky ReLU activation functions exhibit better generalization to the novel 3D dot cloud environment (Figure 5), tolerance to noisy (Figure 6) and sparse optic flow (Figure 7), and robustness in the presence of a moving object (Figure 8) than the networks configured with GELU or Mish. The inclusion of ReLU and leaky ReLU yields improved accuracy in both the CNN and MLP architectures. The estimation of camera translation in the noise test represents an exception—the CNN_RELU and CNN_LEAKY_RELU produce error that is comparable to that of the MLP_GELU and MLP_MISH models (Figure 6d).

### 3.3. Sparseness in the Neural Network Encoding of Optic Flow

It has been proposed that a sparse encoding of data by neural networks confers computational benefits [11,20]. Given that the ReLU activation function is capable of inducing sparse representations [18,20], we investigated whether a sparse encoding of optic flow could explain the advantageous generalization and robustness offered by the neural networks that use ReLU and leaky ReLU activation functions.

#### 3.3.1. Population and Lifetime Sparseness

We used the population and lifetime sparseness metrics to quantify the sparseness of the neural activations produced for optic flow input [38]. Both metrics range between 0 and 1, where values closer to 1 indicate a sparser representation. The population sparseness metric corresponds to the average number of neurons within the same model layer that activate to a specific optic flow sample. On the other hand, the lifetime sparseness metric corresponds to the average proportion of samples in the dataset that activates a particular neuron (see Section 2 for more details).

**Figure 9:**
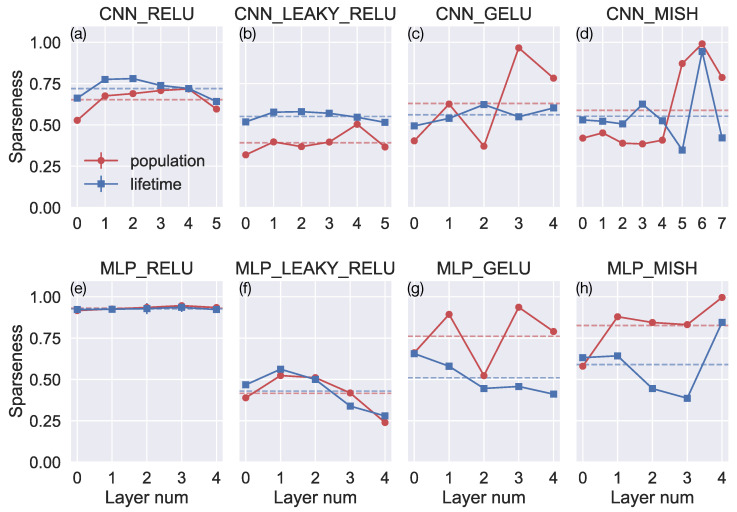
(**a**–**h**) The population (red) and lifetime (blue) sparseness in each layer of the 8 models. Both metrics range between 0 (dense code) and 1 (very sparse code). The red and blue dashed lines indicate the mean population and lifetime sparseness across the network, respectively.

Figure 9 depicts the sparseness metrics computed on the activations within each network layer. Consistent with existing work [20], MLP_RELU produces population and lifetime sparseness values ≈1 in all layers of the network, indicating an incredibly sparse encoding of the optic flow (Figure 9e). The average sparseness metric values (dashed lines) for MLP_RELU are larger than for any other model. CNN_RELU yields smaller sparseness metric values than MLP_RELU, but they are nonetheless larger than those garnered by most of the other networks. It is noteworthy that we obtained similar results when evaluating the sparseness of activations produced for the TR360Cloud.

While these findings support the notion that ReLU acts as a regularizer that promotes a sparse optic flow representation, it is unlikely that sparseness in activations alone plausibly accounts for the favorable accuracy in our generalization and robustness tests. Models with leaky ReLU yield comparable accuracy in these tests (Figure 5, Figure 6, Figure 7 and Figure 8), yet yield some of the smallest sparseness metric values (Figure 9b,f). Moreover, the models with GELU and Mish yield sparseness metric values close to one, particularly in the deep network layers, which exceeds the values associated with CNN_RELU, CNN_LEAKY_RELU, and MLP_LEAKY_RELU models.

**Figure 10:**
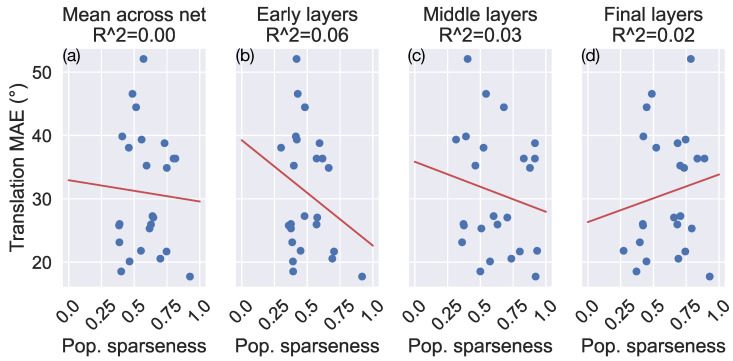
(**a**–**d**) The relationship between population sparseness (x-axis) and MAE obtained when estimating the translational self-motion parameters on the TR360Cloud dataset (y-axis). Plot markers correspond to values obtained from the top 3 networks within each model type. Red lines show the regression curves fitted to the data.

To examine the relationship between sparseness and generalization accuracy across the models more systematically, we fit linear regressions in which the MAE of the estimated camera translation in the TR360Cloud dataset serves as the response variable and the population sparseness metric serves as the predictor variable (Figure 10). We considered MAE as a function of the sparseness averaged across the network (Figure 10a) or averaged within only the early, middle, and final third of the network (Figure 10b–d). We included values from the top 3 networks within each model type rather than only those from the optimal models to increase the number of samples that factor into the analysis. The top 3 networks include the optimal models and the two models that achieve the next lowest validation loss during the hyperparameter search used during model selection (see Section 2). These runner-up networks have the same activation functions as the optimal models but have different hyperparameters. For each regression, we obtain $R^{2}\approx 0$, which indicates that there is no strong association between sparseness and generalization accuracy. We obtained comparable results when the analysis focused on rotation MAE and lifetime sparseness.

#### 3.3.2. Dead Neurons

Table 6 presents the number of dead neurons in each model layer, which offers another way to measure the sparseness of the optic flow signals. Dead neurons never activate to any optic flow sample in the TR360 test set [18]. A large percentage of dead neurons is consistent with a sparse representation, since only a small subpopulation of neurons signals properties of the input data. Table 6 reveals that only the models with ReLU contain dead neurons, and MLP_RELU contains substantially greater proportions (>94% in all but one layer) than CNN_RELU (<7%). Considering that CNN_RELU produces a small percentage of dead neurons and the other models, including those that use leaky ReLU, possess no dead neurons, sparseness from dead neurons cannot account for the robustness of the ReLU and leaky ReLU networks.

#### 3.3.3. Sparseness in Network Weights

The population sparseness, lifetime sparseness, and dead neuron metrics all involve neural activations. This suggests that a sparse set of activations may not account for the resilience of the ReLU and leaky ReLU networks in our robustness tests. Nevertheless, it remains possible that sparseness, in network weights rather than in network activations, could be responsible. To assess the sparseness of network weights, we computed the Sparseness Index (*S*) [39] of each set of weights in the network (see Section 2). Similar to the other sparseness metrics, this metric ranges from 0 to 1. Zero corresponds to a dense uniform weight distribution, while values closer to 1 correspond to a sparse distribution.

**Figure 11:**
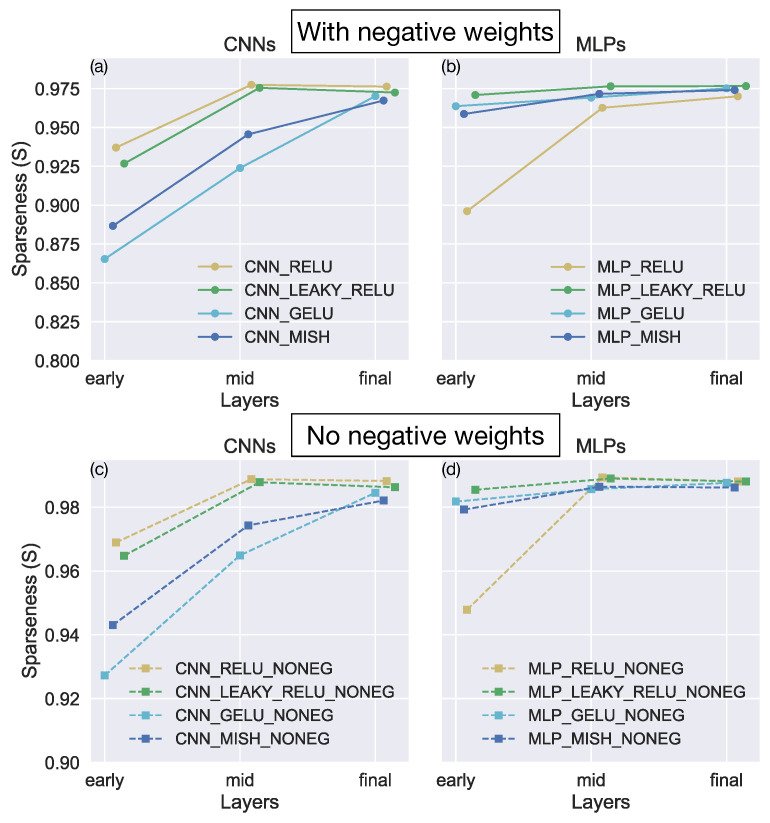
The Sparseness Index (*S*) computed on the weights in the early, middle, or final third of the CNNs (**a**,**c**) and MLPs (**b**,**d**). Solid line demarcates that the analysis includes negative network weights. Dashed line demarcates that the analysis includes only non-negative network weights.

Figure 11a,b shows the Sparseness Index (*S*) averaged across the early, middle, and final thirds of the CNN and MLP network layers, respectively. It is noteworthy that the *S* values indicate that all the models have sparse weight distributions in the final network layers, regardless of activation function. The networks with ReLU and leaky ReLU yield values closest to one, except for MLP_RELU, which generates the lowest *S* values among the MLPs. What could account for the weaker sparseness in the weights of MLP_RELU? One possibility is that the unique rectifying property of ReLU could make it unnecessary for the network to have as substantial sparseness in the weights as the other networks to achieve robust generalization. Because ReLU outputs zeros for all non-positive net inputs, it is sufficient to set many weights to negative values to garner zero output (see Section 4 for extended discussion). If this were the case, MLP_RELU should garner much larger Sparseness Index values if negative weights were excluded from the analysis. Indeed, Figure 11d reveals that this occurs—MLP_RELU produces a comparably large *S* value as CNN_RELU and the networks with leaky ReLU in all but the early layers, where there are few dead neurons (Table 6). Although the Sparseness Index increases in all networks when negative weights are excluded, only MLP_RELU produces a qualitative change in the relative sparseness of the weights.

Overall, our analysis indicates that sparseness in network activations alone cannot explain the favorable accuracy of the models with ReLU and leaky ReLU in the generalization and robustness tests. On the other hand, our analysis indicates that sparseness in weights may be a more plausible factor that contributes to robustness.

### 3.4. Optic Flow Tuning

We sought to gain insight about whether the networks with ReLU and leaky ReLU encode optic flow differently than the other networks. To address this, we focused on characterizing the translation and rotation directions that maximally activate each model neuron in each network layer (i.e., the translation and rotation preferences of each model neuron). For this analysis, we used diagnostic datasets wherein the direction of translation (TestProtocolT) or rotation (TestProtocolR) systematically varied, and we decoded the azimuth and elevation that garners the maximal activation in each model neuron (see Section 2).

#### 3.4.1. Translation Preferences

**Figure 12:**
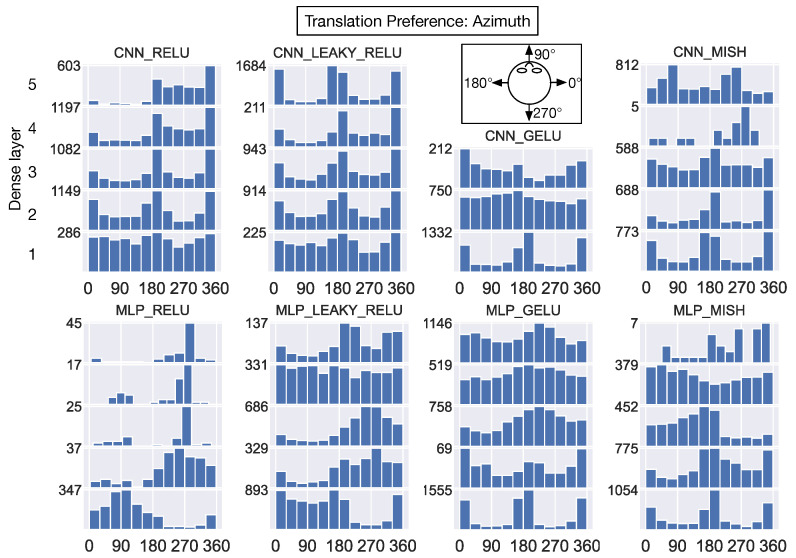
The distribution of translation azimuth angles that yield maximal activation in individual neurons within each dense hidden layer. Each histogram corresponds to the preferences of units in a single model layer, and the histograms associated with the same model are stacked vertically. Histograms assigned smaller “Dense layer” integer labels (top-left panel) correspond to layers earlier in the network, while those with larger integer labels correspond to layers deeper in the network. The x-axis in each histogram corresponds to the preferred translation azimuth angle (0–360°). The y-axis indicates the number of units that possess a particular azimuth angle (bin width: 30°). The schematic atop the 3rd column shows the coordinate system (top-down view).

The histograms in Figure 12 show the translational azimuth angle preference of every neuron in the dense hidden layers of each model. We use a coordinate system wherein the positive and negative x-axis indicates 0° (i.e., rightward translation) and 180° (i.e., leftward translation) azimuths (see top-down schematic view in 3rd column of Figure 12). The CNN_RELU and CNN_LEAKY_RELU exhibit remarkably similar preferred azimuths across the network layers—preferences are broadly distributed in the first hidden layer and become more concentrated at 0°/180° (leftward/rightward) deeper in the networks. The CNN_RELU differs from CNN_LEAKY_RELU in the elevated number of units that prefer backward (270°) self-motion close to the output layer. Interestingly, the first dense hidden layer of the CNNs with smoothly varying negative activations (CNN_GELU and CNN_MISH) yields a bimodal distribution at 0°/180° that resembles the distribution that develops in CNN_RELU and CNN_LEAKY_RELU, but the pattern is not maintained in the deeper layers. The first dense layer of the MLP_RELU and MLP_LEAKY_RELU show a preponderance of units that prefer forward translation (0–180° Figure 12, bottom row). Most units in the next two dense layers of these models demonstrate a preference for backward self-motion (≈270°). This pattern is maintained in the final layers of MLP_RELU but not in MLP_LEAKY_RELU.

**Figure 13:**
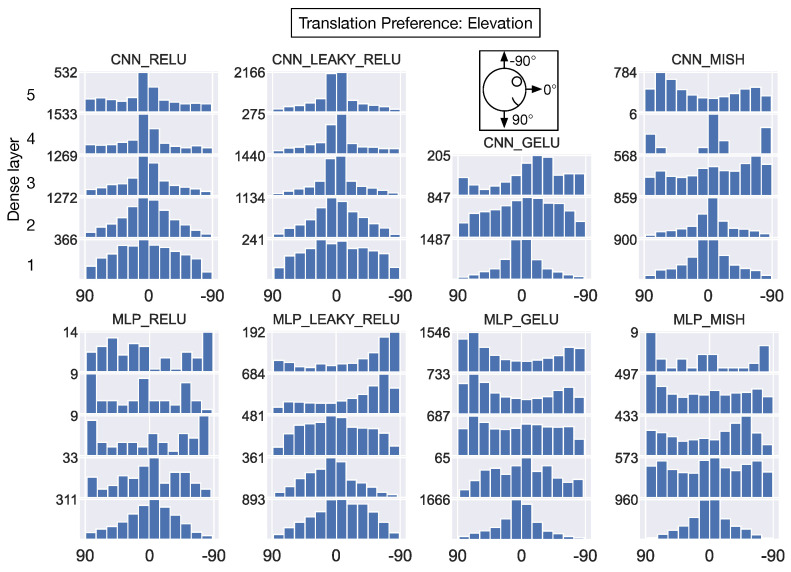
The translation elevation angle preference of individual neurons within each model dense hidden layer. Same format as Figure 12. The schematic atop the 3rd column shows the coordinate system (side view).

With respect to translation elevation angle preferences (Figure 13), all the networks demonstrate remarkable consistency in the dominant preference for 0° elevations (parallel to the ground) within the first hidden layer. This peak at 0° persists across layers only in the CNN_RELU, CNN_LEAKY_RELU, and CNN_GELU models. In most of the other networks, a predominance of units develop preferences for either ≈90° (downward translation) or ≈−90° (upward translation).

#### 3.4.2. Rotation Preferences

**Figure 14:**
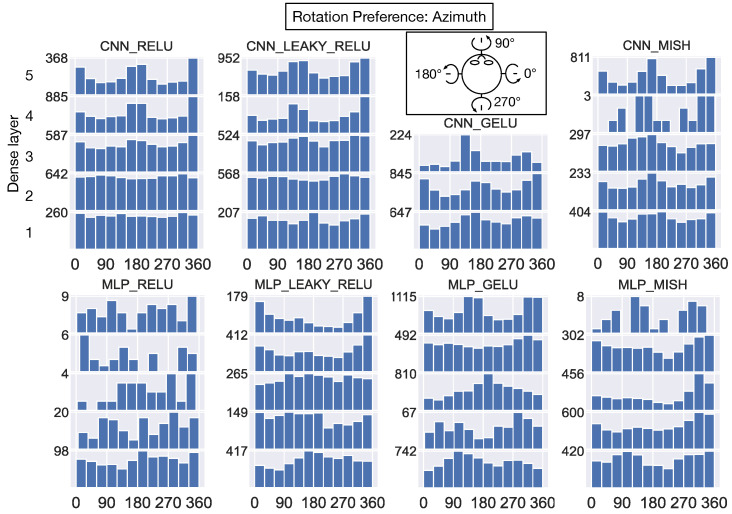
The distribution of preferred rotation azimuth angles of individual neurons within each model dense hidden layer. Same format as Figure 12.

**Figure 15:**
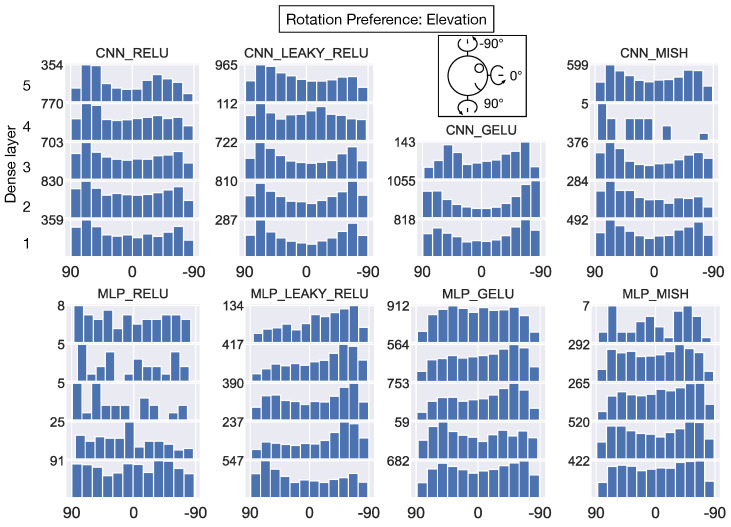
The distribution of preferred rotation elevation angles of individual neurons within each model dense hidden layer. Same format as Figure 12.

Next, we characterized the distribution of rotation preferences in the model layers. Figure 14 focuses on the azimuth angle of the preferred direction of rotation (see top-down schematic view in 3rd column of Figure 14). Interestingly, the rotation azimuth angle preferences are broadly distributed in the first dense hidden layer of all the networks. Similar to the distribution of translation azimuth preferences that emerge in deeper layers of CNN_RELU and CNN_LEAKY_RELU (Figure 12), bimodal distributions that peak at ≈0/180° appear in the deeper layers of CNN_RELU, CNN_LEAKY_RELU, CNN_MISH, and MLP_GELU for rotation azimuth (Figure 14). Figure 15 reveals a similar pattern where the preference rotation elevation angle is broadly distributed in the first dense layer, and units in most networks cluster in their elevation preferences in the deeper layers. Many units in these deeper layers demonstrate a preference for rotation about either ≈90°, ≈−90°, or both elevations.

Taken together, CNN_RELU and CNN_LEAKY_RELU exhibit highly similar distributions of translation and rotation preferences across the network layers. The MLP variants with the same ReLU and leaky ReLU activation functions demonstrate consistency neither with the CNN variants nor amongst themselves. For translation (elevation) and rotation (azimuth and elevation), neurons in most models exhibit similar preferences in first dense hidden layer, and 1-2 dominant directions tend to emerge by the final layers.

## 4. Discussion

Despite its relative simplicity, the ReLU activation function has been used in numerous state-of-the-art CNNs for image recognition over the past decade [47,48,49]. While transformer-based large language models (LLMs) [50,51] have popularized the GELU activation function, recent studies nevertheless demonstrate benefits of ReLU [52,53]. Our work characterizes how ReLU shapes the accuracy and encoding of optic flow compared to other activation functions. While all the networks accurately estimate the self-motion of the camera from novel optic flow patterns generated during movement through the environments encountered in training (translation: <6.0° MAE; rotation: <0.3°/s MAE), we found that those that use the ReLU and leaky ReLU activation functions performed this task with substantially less error on optic flow generated from a new environment. These networks also demonstrate superior robustness in our experiments when we added noise, increased sparseness, and introduced an independent moving object into the optic flow. The MLP configured with ReLU achieves this with astonishingly sparse optic flow encoding—>94% neurons never activated to any of the test optic flow samples in all but the first hidden layer (Table 6). According to the dead neuron metric, this corresponds to 78.4% average sparseness across all the network layers. Interestingly, this average sparseness falls within the 68-84% range obtained from 3-layer MLPs that use ReLU when trained on MNIST, CIFAR10, and other image datasets [20]. It is striking that the average sparseness in our MLP is similar to the ≈80% sparseness that Glorot and colleagues found that is associated with the lowest test error on the MNIST dataset. This suggests a consistent effect of sparseness on generalization across image and optic flow data, at least in MLP networks with ReLU. It is noteworthy that MLP_RELU achieves this high level of sparseness without any explicit regularization. Despite having the same activation function, the CNN_RELU model yields vastly different percentages of dead neurons within its layers (3.0% vs. 78.4% average sparseness; see Table 6). This suggests that the convolutional and max pooling layers substantially influence the encoding of optic flow. Indeed, the distributions of translation and rotation preferences of individual neurons in the MLP_RELU and CNN_RELU are substantially different (Figure 12, Figure 13, Figure 14 and Figure 15; leftmost column).

Despite the substantially different percentages of dead neurons, the MLP_RELU and CNN_RELU networks have large average population and lifetime sparseness (Figure 9). Given that these networks yield the lowest error in our generalization and robustness tests, this may seem to indicate a positive correlation between sparseness and generalization accuracy. However, when taking all the simulated networks into account, we observe no systematic relationship (Figure 10). Indeed, Figure 10 reveals considerable variability in the mean error obtained in networks with similar levels of sparseness. This is consistent with the fact that the networks with leaky ReLU yield similar generalization accuracy and robustness to those that use ReLU (Figure 5, Figure 6, Figure 7 and Figure 8), despite having relatively low average population and lifetime sparseness (Figure 9).

What could account for the superior generalization and robustness of the networks with ReLU and leaky ReLU? One possibility is an optic flow coding strategy in which these networks set a larger number of weights to 0 than the networks that use GELU and Mish (Figure 11). Such a sparse distribution could promote robustness because small perturbations to the input may not change the output much if the input recruits the same small set of nonzero weights with or without the perturbation [11,20,22,23,24]. Our analysis suggests that similarly high degrees of sparseness in network weights may nevertheless give rise to a range of sparseness in activations (Figure 9, Table 6). For example, CNN_RELU and CNN_LEAKY_RELU possess similar weight sparseness values yet yield more dissimilar population and lifetime sparseness values. It is noteworthy that while CNN_RELU and CNN_LEAKY_RELU demonstrate consistency in translation and rotation preferences of neurons within the network layers, the MLPs do not. This suggests that a range of translation and rotation preferences could be associated with robust generalization, at least between network architectures.

Interestingly, the MLP_RELU model may employ a different coding strategy than the other networks, given its relatively low weight sparseness. Due to its unique rectifying behavior on negative inputs, the network has more options at its disposal to implement a sparse encoding of optic flow. Unlike the other networks that must either set weights to exactly 0 or balance the magnitude of positive and negative weights to induce a sparse code, setting many weights to negative values is sufficient for generating a sparse code since the ReLU activation will suppress any negative net input. Our findings suggest that ReLU allows the network to use large negative weights to induce silent model units with zero activation, resulting in a sparse encoding of optic flow.

## 5. Conclusions

The present work characterizes the accuracy, robustness, and encoding of optic flow in different CNN and MLP neural networks. Models with the ReLU and leaky ReLU activation functions offer superior generalization and robustness when estimating self-motion from optic flow compared to networks with the smoother GELU and Mish activation functions. Our results support the notion that this difference in performance stems from sparseness in the network weights, but not in network activations. Future studies should explore whether ReLU and leaky ReLU may offer similarly favorable performance on other navigation-related tasks, such as estimating relative object motion, depth, and structure from motion.

## Figures and Tables

**Table 1:** Optic flow dataset specifications. Translation and rotation are denoted by T and R, respectively, while TR refers to optic flow derived from self-motion involving both translation and rotation. Only TR360 is used to train the models (fit weights), and the train/test/val sizes are listed. Straight-ahead heading corresponds with azimuth and elevation angles of 0°. Table adapted and modified with permission from Table 1 in [17].

Dataset	Description	Size (Num Samples N)	Independent Variables
TR360	Simulated self-motion toward either a frontoparallel plane (wall) or above a ground plane. T and R direction is uniform random: TR elevation [−180, 180]°, TR azimuth [−90, 90]°.	total: 12,060 (6030 frontoparallel + 6030 ground) train: 6030 validation: 3015 test: 3015	T speed: [0.5, 1.0, 1.5] m/s R speed: [0, 5, 10] °/s Frontoparallel plane depth: [2, 4, 8, 16, 32] m
TR360Cloud	Same as TR360, except self-motion is simulated through a 3D cloud of dots. Depth of each dot is uniform random: [2, 32] m.	3015 test	T speed: [0.5, 1.0, 1.5] m/s R speed: [0, 5, 10] °/s
TestProtocolT	Diagnostic set of optic flow patterns used to evaluate neural tuning to specific T directions	514 (512 combinations of T azimuth and elevation & ±90° vertical)	T azimuth: [0, ±11.25, ±22.5, …, ±180]° T elevation: [0, ±11.25, ±22.5, …, ±90]°
TestProtocolR	Diagnostic set of optic flow patterns used to evaluate neural tuning to specific R directions	514 (512 combinations of R azimuth and elevation & ±90° vertical)	R azimuth: [0, ±11.25, ±22.5, …, ±180]° R elevation: [0, ±11.25, ±22.5, …, ±90]°

**Table 2:** Ranges used in random search for optimal neural network hyperparameters. Except for the learning rate and α, hyperparameters were selected on a per-layer basis on every iteration of the search. The hyperparameter α was only involved when optimizing the networks configured with the leaky ReLU activation function. The * symbol indicates that the learning rate and α values were drawn randomly from the indicated set.

Hyperparameter	Value Range
Number of convolution and max pooling stacks	[1, 3]
Number of dense layers	[1, 5]
Number of convolutional filters	[2, 300]
Number of dense units	[2, 10,000]
Convolutional unit filter size	[2, 15]
Max pooling window size	[2, 4]
Max pooling stride length	[1, 3]
Learning rate	[$1\times 10^{-5}$, $1\times 10^{-4}$, $1\times 10^{-3}$, $1\times 10^{-2}$] *
Leaky ReLU activation function α	[0.01, 0.1, 1, 2] *

**Table 3:** Optimized CNN hyperparameters. Entries in lists correspond to the values in each relevant layer of the network. For example, [104, 213] means that there are 104 filters in the first convolutional layer and 213 in the second.

Hyperparameter	CNN_RELU	CNN_LEAKY_RELU	CNN_GELU	CNN_MISH
Number of convolution and max pooling stacks	1	1	2	3
Number of dense layers	5	5	3	5
Number of convolutional filters	[157]	[105]	[104, 213]	[105, 112, 279]
Number of dense units	[2997, 7566, 5979, 6709, 2631]	[2073, 6418, 5792, 1020, 8613]	[6305, 7621, 2990]	[4796, 3330, 6632, 411, 5166]
Convolutional unit filter size	[2]	[2]	[2, 2]	[2, 2, 2]
Max pooling window size	[2]	[2]	[4, 4]	[3, 3, 3]
Max pooling stride length	[3]	[2]	[1, 2]	[1, 3, 1]
Learning rate	$1\times 10^{-5}$	$1\times 10^{-5}$	$1\times 10^{-4}$	$1\times 10^{-4}$

**Table 4:** Optimized MLP hyperparameters. Same format as in Table 3.

Hyperparameter	MLP_RELU	MLP_LEAKY_RELU	MLP_GELU	MLP_MISH
Number of dense layers	5	5	5	5
Number of dense units	[2958, 6244, 3234, 5067, 2651]	[7628, 4705, 8044, 6970, 2027]	[6783, 502, 6201, 5015, 9342]	[4371, 5412, 3814, 6625, 3240]
Learning rate	$1\times 10^{-3}$	$1\times 10^{-4}$	$1\times 10^{-4}$	$1\times 10^{-4}$

**Table 5:** Number of parameters learned in each neural network model.

Model	Number of Parameters
CNN_RELU	137,480,586
CNN_LEAKY_RELU	75,904,186
CNN_GELU	104,532,948
CNN_MISH	48,476,092
MLP_RELU	69,846,657
MLP_LEAKY_RELU	147,374,365
MLP_GELU	87,565,173
MLP_MISH	93,013,120

**Table 6:** The number and percentage of dead neurons in each layer of each neural network. Entries in lists represent the value in corresponding layers of the network.

Network Model	Number of Dead Neurons	Percentage of Neurons Dead
CNN_RELU	[0/157, 20/2997, 121/7566, 258/5979, 323/6709, 179/2631]	[0.0%, 0.67%, 1.60%, 4.32%, 4.81%, 6.80%]
CNN_LEAKY_RELU	[0/105, 0/2073, 0/6418, 0/5792, 0/1020, 0/8613]	[0.0%, 0.0%, 0.0%, 0.0%, 0.0%, 0.0%]
CNN_GELU	[0/104, 0/213, 0/6305, 0/7621, 0/2990]	[0.0%, 0.0%, 0.0%, 0.0%, 0.0%]
CNN_MISH	[0/105, 0/112, 0/279, 0/4796, 0/3330, 0/6632, 0/411, 0/5166]	[0.0%, 0.0%, 0.0%, 0.0%, 0.0%, 0.0%, 0.0%, 0.0%]
MLP_RELU	[224/2958, 5903/6244, 3144/3234, 4975/5067, 2505/2651]	[7.57%, 94.54%, 97.22%, 98.18%, 94.49%]
MLP_LEAKY_RELU	[0/7628, 0/4705, 0/8044, 0/6970, 0/2027]	[0.0%, 0.0%, 0.0%, 0.0%, 0.0%]
MLP_GELU	[0/6783, 0/502, 0/6201, 0/5015, 0/9342]	[0.0%, 0.0%, 0.0%, 0.0%, 0.0%]
MLP_MISH	[0/4371, 0/5412, 0/3814, 0/6625, 0/3240]	[0.0%, 0.0%, 0.0%, 0.0%, 0.0%]

## Data Availability

The trained models and datasets are available on Hugging Face: https://huggingface.co/collections/OWLab/optic-flow-cnns-mlps-15x15-673f74ad3d22cacbcc19c39c (accessed on 21 November 2024). Code is available on GitHub: https://github.com/owlayton/DL-ActFuns-Acc-SelfMotion-Release (accessed on 21 November 2024).

## Abbreviations

The following abbreviations are used in this manuscript:

CNN	Convolutional neural network
MLP	Multi-layer perceptron neural network
ReLU	Rectified linear unit activation function
GELU	Gaussian error linear unit activation function
MSE	Mean squared error
MAE	Mean absolute error
LLM	Large language model
